# Optic-null space medium for cover-up cloaking without any negative refraction index materials

**DOI:** 10.1038/srep29280

**Published:** 2016-07-07

**Authors:** Fei Sun, Sailing He

**Affiliations:** 1 Centre for Optical and Electromagnetic Research, State Key Laboratory of Modern Optical Instrumentation, Zhejiang Provincial Key Laboratory for Sensing Technologies, East Building #5, Zhejiang University, Hangzhou 310058, China; 2 Department of Electromagnetic Engineering, JORCEP, School of Electrical Engineering, Royal Institute of Technology (KTH), S-100 44 Stockholm, Sweden

## Abstract

With the help of optic-null medium, we propose a new way to achieve invisibility by covering up the scattering without using any negative refraction index materials. Compared with previous methods to achieve invisibility, the function of our cloak is to cover up the scattering of the objects to be concealed by a background object of strong scattering. The concealed object can receive information from the outside world without being detected. Numerical simulations verify the performance of our cloak. The proposed method will be a great addition to existing invisibility technology.

Invisibility is a special kind of optical illusion that can conceal the target object to outside viewers. With the development of new theory (e.g. transformation optics (TO)^1^,^2^,^3^) and new technologies (e.g. meta-materials^4^,^5^), many different schemes have been proposed to design invisibility cloaking, some of which have also been experimentally demonstrated for some specific frequencies^6^,^7^,^8^,^9^,^10^,^11^,^12^,^13^,^14^,^15^. These methods are based on different principles, which can mainly be classified into two kinds: one is optical-isolated cloaking (e.g. point-extended cloaking by TO^6^,^7^, or cloaking based on the geometrical optic^8^,^9^); the other is scattering cancellation cloaking (e.g. the complementary medium method^10^,^11^, cloaking based on optimization algorithms^12^,^13^, and active cloaking^14^,^15^). However, the current results are still far from true invisibility.

In 2014, we proposed another scheme to achieve invisibility, namely, the scattering cover-up method^16^. Compared to other methods, ours can achieve the following simultaneously: Firstly, the concealed object can see the outside world without being seen, secondly, when some parameter (e.g. size, shape, and position) of the concealed objects changes, the cloak still works, and thirdly, there is no need to know or detect any information about the detecting wave in advance. With this method, the concealed object should be set beside a background object with large scattering. To an outside viewer, the scattering of the object to be concealed is covered up by the strong scattering of the background object. However, we need negative refraction index materials to realize this scattering cover-up cloaking due to the space folding transformation, which makes it difficult to be realized experimentally.

The optic-null medium (ONM) is a special kind of high anisotropic homogenous medium without any negative component, which has been experimentally demonstrated for hyper-lenses^17^,^18^ and concentrators^19^. Combined with the theory of optical surface transformation (OST)^20^,^21^, ONM has also been utilized to design many other novel optical devices, including wave front reshapers^20^, zero-order resonators^22^, and overlapping optical illusions without any negative refraction index materials^23^. The ONM is obtained by extreme space-extension transformations on a surface in the reference space to a volume in the real space, which leads to a highly anisotropic medium in the real space. In this study, we found that such an ONM can be utilized to achieve the scattering cover-up cloaking without any negative refraction index materials.

## Method and Results

**Figure 1:**
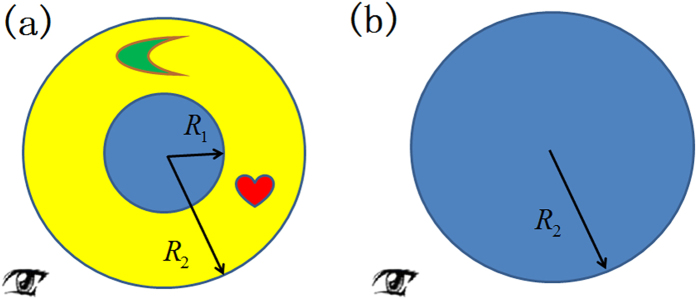
The basic scheme for the scattering cover-up cloaking by ONM. (**a**) The background object (blue cylindrical PEC with a radius *R*_1_), the concealed objects (green moon and red heart), and the cloaking with a radius *R*_2_ (yellow ONM with main axis along radial direction). (**b**) A PEC cylinder with radius *R*_2_. This figure is drawn by one author (Fei Sun).

Figure 1 shows the basic scheme of our method. In the real space, we have a cylindrical perfect electric conductor (PEC) with a radius *R*_1_ (colored blue) in the center, wrapped by ONM with the main axis along the radial direction with a radius *R*_2_ (colored yellow). The concealed objects are set inside the ONM (e.g. the green moon and the red heart). We should note that these concealed objects can be filled with any materials of arbitrary shapes at any position inside the ONM. To the outside viewer, the scattering field of the whole system in Fig. 1(a) is nearly the same as the scattering pattern of a cylindrical PEC with radius *R*_2_ in Fig. 1(b).

Unlike the previous method (i.e. the space folding transformation) to achieve the scattering cover-up^16^, our scheme based on the ONM has unique features. Firstly, we do not need any negative refraction index materials, as no space folding transformation is utilized. Secondly, the concealed objects and the real background object are wrapped inside the cloak. Note that the background object is outside the cloak in previous method^16^. Thirdly, we should emphasize that we have two background objects of different functions in this scheme. The PEC cylinder of a radius *R*_1_ in Fig. 1(a) is the real background object. The PEC cylinder with a radius *R*_2_ in Fig. 1(b) is the effective background object. If we put a real background object in Fig. 1(a), the outside viewer can only see the effective background object in Fig. 1(b) even if some additional objects are introduced inside the cloak (the yellow shell).

**Figure 2:**
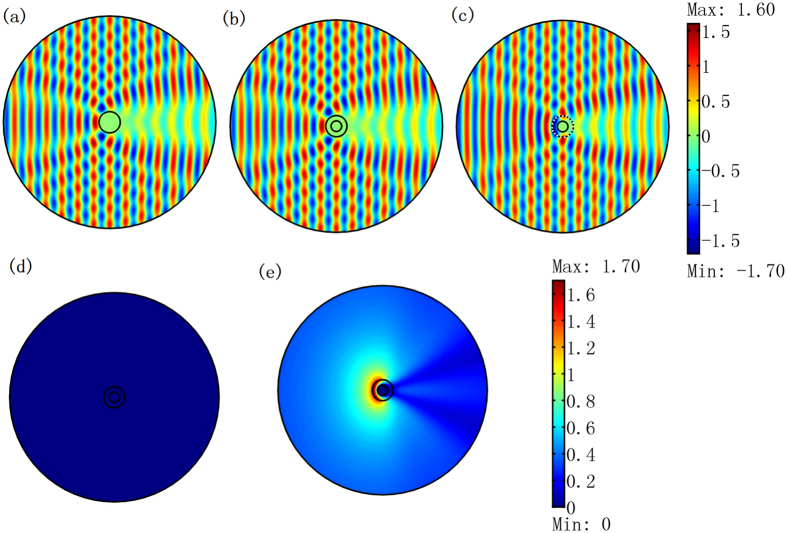
2D FEM simulation results for a TE wave case. The incident wave is a plane wave from the left to the right. (**a**–**c**) are the total electric field distribution. (**a**) Only a PEC cylinder with radius *R*_2_ = 2λ_0_/3. (**b**) A PEC cylinder with a radius *R*_1_ = λ_0_/3 wrapped by the ONM given by Eq. (1) with a radius *R*_2_. (**c**) Only a PEC cylinder with a radius *R*_1_. (**d**) The absolute value of the difference between the fields in (**a**,**b**). (**e**) The absolute value of the difference between the fields in (**a**,**c**).

The relative permittivity and permeability of an ONM with main axis along the radial direction can be expressed by:


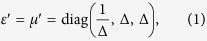


where Δ is an extremely small positive value. From the perspective of OST, the surfaces *r*′ = *R*_1_ and *r*′ = *R*_2_ connected by the ONM with main axis along the radial direction are equivalent surfaces. This means that a PEC cylinder with radius *R*_1_ wrapped by such ONM with radius *R*_2_ in the real space performs equivalently to a PEC cylinder with a radius *R*_2_ in the reference space (i.e. the scattering field of the two systems are identical). Numerical simulations based on finite element method (FEM) verify this idea (see Fig. 2). It can be seen that the electric field distributions for the case consisting of only a PEC cylinder of a radius *R*_2_ and the case consisting of a PEC cylinder of a radius *R*_1_ wrapped by the ONM described by Eq. (1) of a radius *R*_2_ are exactly the same (see Fig. 2(a,b)), which is different from the case consisting of only a PEC cylinder of a radius *R*_1_ without any ONM shell (see Fig. 2(c)). Note that our simulation is based on COMSOL Multiphysics throughout the paper.

If we set some other additional objects to be concealed inside the ONM as shown in Fig. 1(a), will the scattering field distribution of the whole system still be the same as the one in Fig. 1(b)? Numerical simulation results show that even if we set some additional objects inside the ONM in Fig. 1(a), the scattering of the whole system is still nearly the same as the case of the PEC cylinder in Fig. 1(b). If the additional objects are PEC, the scattering is the same as the case in which only the background object is present. If the additional objects are dielectric objects, the scattering is slightly changed but still nearly the same as the case that only background object exists (we will explain this in the discussion).

**Figure 3:**
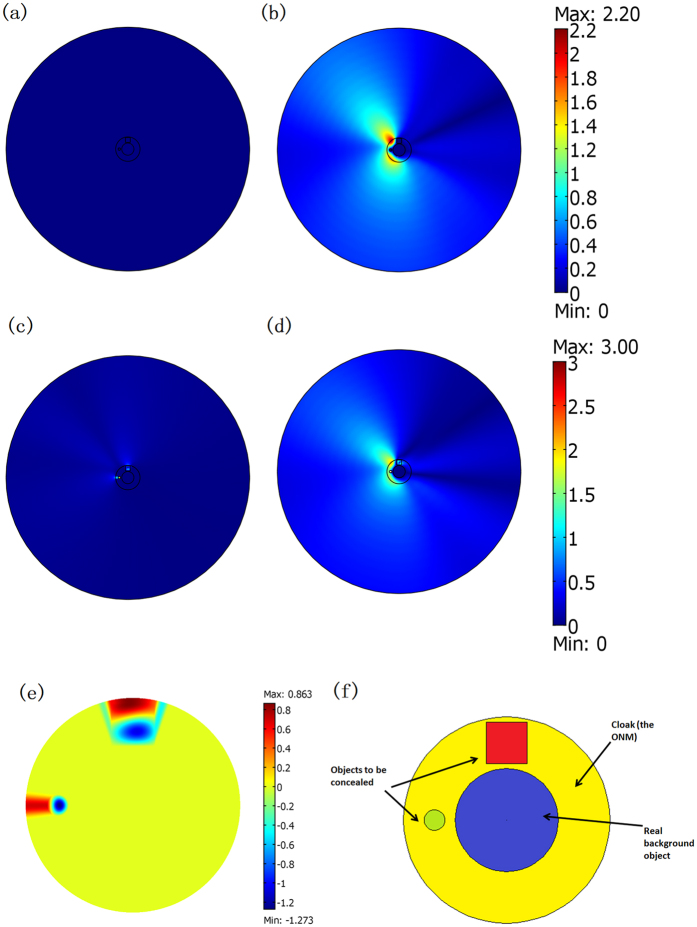
2D FEM simulation results for a TE wave case. The incident wave is a plane wave from the left to the right. (**a**–**d**) We only plot the absolute value of the field difference as compared to the case that only the background object exists in Fig. 2(a). (**a**,**b**) The concealed objects are PEC cylinders (one is the circular column on the left and the other is the rectangular column on the top). (**c**,**d**) The left cylinder is filled by medium for which *ε*_r_ = 1 and *μ*_r_ = 40, and for upper rectangular cylinder *ε*_r_ = 10 and *μ*_r_ = 1. (**a**,**c**) The whole system is wrapped by the cloak (i.e. the ONM described by Eq. (1)). (**b**,**d**) The whole system is not wrapped by the cloak. (**e**) The total electric field within the cloak in (**c**) is zoomed in. (**f**) The shapes and positions of the concealed objects relative to the cloak and background object in this example. The sizes of the concealed objects on the left and top are 0.067λ_0_ and 0.267λ_0_, respectively, for the radius and the side length. The white region means that the value is beyond the color bar.

As shown in Fig. 3, the background object and the cloak have the same geometrical shape and size as those in Fig. 2. The concealed objects here are a cylinder of a radius λ_0_/15 on the left side of the background object and a rectangular cylinder of a side length 4λ_0_/15 on top of the background object. The total electric field distributions in Fig. 3(a,c) are nearly the same as the field distribution in Fig. 2(a), which shows that such a scheme can be utilized via scattering cover-up cloaking.

To describe the scattering cover-up effect quantitatively, we use the averaged relative change of the scattering field γ, which is defined by:


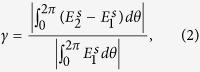


where *E*_1_^s^ is the scattered far field when only the effective background object (i.e. a PEC cylinder of a radius *R*_2_ in Fig. 2(a)) exists. *E*_2_^s^ is the scattered far field of the whole system (i.e. some additional objects are set aside the real background object). The integration is calculated on a large circle (i.e. theta varies from 0 to 2π) in the far field. A smaller *γ* means the cloaking effect is better.

For the cases with and without cloaking in Fig. 2(b,c), we have *γ* = 0.0032% and 124.3%, respectively, when there is no object to be concealed. This indicates that the cloak works well. We then check whether this cloak can cover up the objects we want to conceal in the neighborhood. If the concealed objects are PECs, we have *γ* = 0.0028% and 113.1%, with and without the cloak, in Fig. 3(a,b), respectively. If the concealed objects are dielectric media, we have *γ* = 14.4% and 116.4%, with and without the cloak, in Fig. 3(c,d), respectively. We can see that the cloaking effect is better if the concealed objects are PEC. If the concealed objects are dielectric media, our cloak still has a certain scattering cover-up effect compared to the case when the cloak is removed.

Another thing we should like to mention is the concealed region can still receive the electromagnetic wave from the outside world without being detected (e.g. for the case that the concealed objects are dielectric media in Fig. 3(c)). To see this clearly, we only plot the total electric field distribution within the ONM of the Fig. 3(c) in Fig. 3(e). It shows that the electric field indeed enters the concealed region, which means that the cloaked objects can see the outside world without being seen by our scheme.

**Figure 4:**
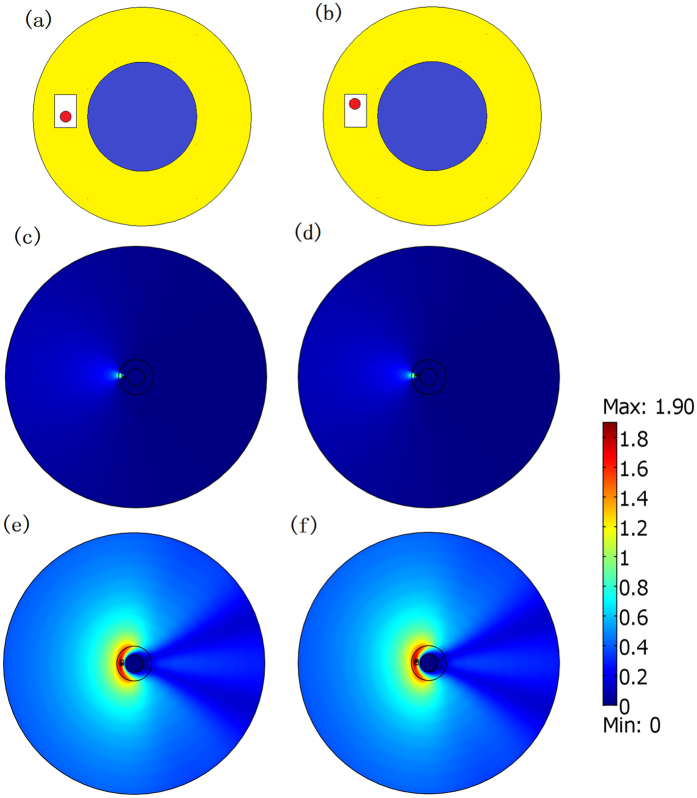
(**a**,**b**) Show the relative position of the cloak (yellow region), the background object (blue cylinder), the air region (white rectangle), and the concealed cylinder with *ε*_r_ = 5 and *μ*_r_ = 1 (red circle). (**c**–**f**) Are 2D FEM TE wave simulation results of the absolute value of the field difference as compared to the case that only the background object exists in Fig. 2(a). The center objects in (**c**,**d**) correspond to objects plotted in (**a**,**b**), respectively. In (**e**,**f**), we simply remove the cloak (the yellow shell) while keeping other objects unchanged in (**a**,**b**), respectively.

Next, we change the positions and geometrical shapes of the concealed objects to check weather our cloaking still works. As shown in Fig. 4, even if the positions and shapes of the concealed objects change, the cloak can still help to cover up their scattering pattern by the strong scattering of the background object in the center. In Fig. 4, we have a rectangular region filled by air within our cloak. A circular cylinder (*ε*_r_ = 5 and *μ*_r_ = 1) is set inside the air rectangle at different positions in Fig. 4(a,b), which means that the concealed object can move freely within the concealed region. As shown in Fig. 4(c,d), our cloak still works (i.e. the scattering pattern of the whole system is still nearly the same as the case consisting of only the background object in Fig. 2(a)). We have *γ* = 8.7% and 8.8%, respectively, in Fig. 4(c,d). For comparison, the field distributions for the case in which the cloak is removed in Fig. 4(a,b) are also plotted in Fig. 4(e,f), where *γ* = 91.2% and 91.2%, respectively.

## Discussion

**Figure 5:**
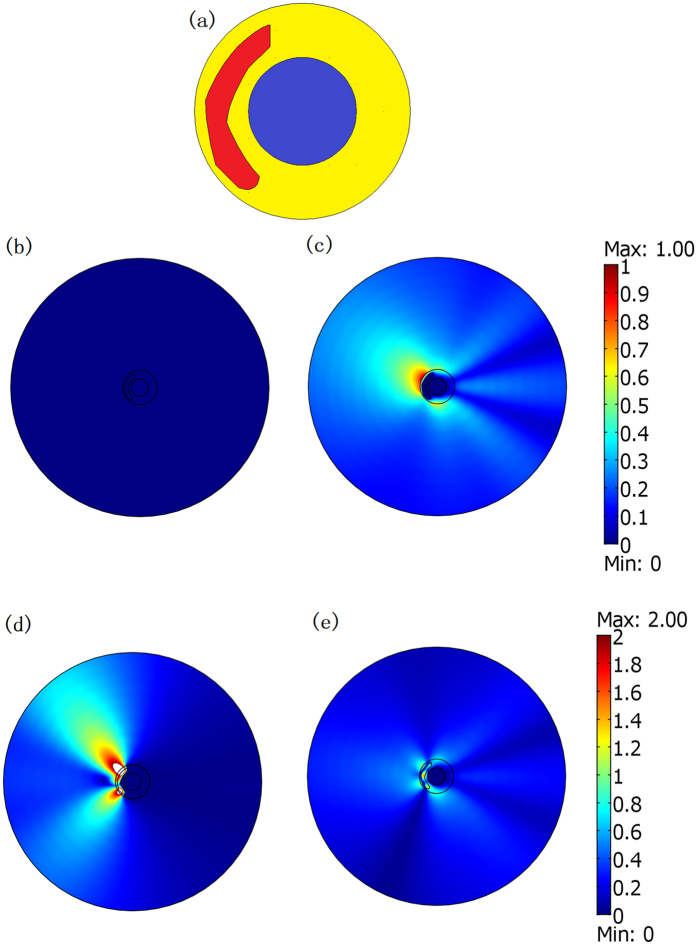
(**a**) Shows the shape of the concealed region (colored red) in this simulation. The yellow and blue regions still stand for the cloak and the background object, respectively. (**b**–**e**) Are 2D FEM simulation results for a TE wave case. We plot the absolute value of the field difference as compared with the case that only the background object exists in Fig. 2(a). (**b**,**c**) Are the cases where the red region is set by the PEC with and without the cloak (*γ* = 0.00076% and 42.9%), respectively. (**d**,**e**) Are the cases where the red region is set by the dielectric medium (*ε*_r_ = 5 and *μ*_r_ = 1) with and without the cloak (*γ* = 68.7% and 43.5%), respectively.

If the concealed region is PEC, it can be designed with an arbitrary shape. This can be easily understood from the perspective of the OST. Whatever the shape and location of the PEC inside the ONM, the whole system will perform like a PEC with the same boundary as the outside boundary of the ONM, as all boundaries connected by the ONM are equivalent boundaries. In Fig. 5, we choose a much bigger irregular PEC within the cloak. The numerical simulation in Fig. 5(b) shows that the field distribution in this case is still the same as the field distribution when only the background object exists in Fig. 2(a). To achieve a scattering cover-up effect, the size of the hided PEC object should be smaller than the size of the virtual background object.

However, if the concealed object is dielectric medium with a relatively larger size, the cloaking effect of the ONM will be greatly influenced. From the perspective of the OST, the whole system now performs like a cylinder with a cut angle filled by some other dielectric materials (i.e. the extension of the previous dielectric materials). If the region of the dielectric materials inside the cloak is not too large (i.e. the cut angle is not very large), the scattering of the whole system is still nearly the same as a background PEC cylinder without any cut angles. However, if the region of the dielectric materials inside the cloak is much larger, the scattering of the whole system will obviously deviate from the scattering of the background object (see Fig. 5(d)).

If the concealed objects are PEC, the cloaking effect is perfect in theory. In this case, the direction of the incident wave will not influence the performance of the proposed cloak. If the concealed objects are dielectric, the cloaking effect is not perfect in theory. Now the performance of the cloak will be influenced by many factors, e.g. the direction and wavelength of the incident wave, the size and shape of the hided object.

## Conclusion

A new way to achieve invisibility via ONM has been explored in this study: namely, the scattering cover-up method. In this method, we have used a background object of strong scattering wrapped by the ONM to cover up the weak scattering of the objects to be concealed (also inside the ONM). Compared to previous invisibility cloaking methods designed by TO, the proposed method has many advantages. The concealed objects can move freely inside the cloak and can receive information from the outside world without being detected. The proposed cloak can be achieved without any negative refraction index materials and without knowing any information about the detecting wave in advance.

## Additional Information

**How to cite this article**: Sun, F. and He, S. Optic-null space medium for cover-up cloaking without any negative refraction index materials. *Sci. Rep.* **6**, 29280; doi: 10.1038/srep29280 (2016).
